# Effect of anticoagulant/antiplatelet therapy on the development and progression of diabetic retinopathy

**DOI:** 10.1186/s12886-022-02323-z

**Published:** 2022-03-17

**Authors:** Chi-Juei Jeng, Yi-Ting Hsieh, Cheng-Li Lin, I-Jong Wang

**Affiliations:** 1 Department of Ophthalmology, Taipei Medical University-Shuang-Ho hospital, Ministry of Health and Welfare, New Taipei City, Taiwan; 2 Graduate Institute of Clinical Medicine, College of Medicine, National Taiwan University, Taipei, Taiwan; 3 Department of Ophthalmology, School of Medicine, National Taiwan University Hospital, Taipei, Taiwan; 4 Management Office for Health Data, China Medical University, Taichung, Taiwan; 5 Graduate Institute of Clinical Medical Science, China Medical University, Taichung, Taiwan

**Keywords:** Diabetic retinopathy, Anticoagulant, Antiplatelet, Aspirin

## Abstract

**Background:**

We investigated whether antiplatelet/anticoagulant (APAC) therapy can protect patients with type 2 diabetes mellitus (T2DM) from the development or progression of diabetic retinopathy (DR).

**Methods:**

This is a retrospective cohort study using Longitudinal Health Insurance Database in Taiwan. A total of 73,964 type 2 diabetic patients older than 20 years old were included. Hazard ration (HR) of non-proliferative DR (NPDR), proliferative DR (PDR), and diabetic macular edema (DME) were analyzed with APAC usage as a time-dependent covariate. Age, sex, comorbidities, and medicines were further adjusted in a multi-variable model. Contributions of respective APAC was investigated with sensitivity analysis.

**Results:**

Compared with nonusers, APAC users had a lower cumulative incidence of NPDR (*P* < 0.001), overall incidence of NPDR (10.7 per 1000 person-years), and risk of developing NPDR (adjusted HR = 0.78, 95% CI = 0.73–0.83). However, no significant differences were observed between APAC users and nonusers in the risks of PDR or DME. Hypertension, diabetic nephropathy and diabetic neuropathy were risk factors for NDPR development, while heart disease, cardiovascular disease, peripheral arterial occlusive disease, and statin usage were covariates decreasing NPDR development. Aspirin and Dipyridamole showed significant protection against NPDR development. Clopidogrel, Ticlopidine, and warfarin showed enhanced protection in combination with aspirin usage.

**Conclusions:**

APAC medications have a protective effect against NPDR development. Diabetic patients benefit from single use of aspirin or dipyridamole on prevention of NPDR.

**Supplementary Information:**

The online version contains supplementary material available at 10.1186/s12886-022-02323-z.

## Background

Diabetes mellitus (DM) is characterized by chronic hyperglycemia, which not only causes metabolic disorder but also has profound effects leading to macro- and micro-vasculopathies [1]. Hyperglycemia not only disturbs vascular homeostasis, induces inflammation, endothelial dysfunction, and platelet hyperactivitiy [2], but also causes imbalances of glucose and insulin levels, leading to a procoagulative state [3]. Therefore, patients with DM are at a high risk of thrombosis, which causes diseases such as atherosclerosis [4]. Up to 80% of deaths from type 2 diabetics mellitus (T2DM) are thrombotic [5], and 75% of such deaths result from cardiovascular events [6], with the others resulting from complications of peripheral vascular diseases [7].

Accordingly, antiplatelet/anticoagulant (APAC) therapy with medications, such as low-dose aspirin, is used to target one or multiple pathways responsible for accelerating atherosclerosis and its thrombotic complications in diabetic patients [8]. However, the costs and benefits of primary cardiovascular prevention with aspirin have long been debated. Antithrombotic Trialists’ (ATT) Collaboration reported that aspirin yielded 12% proportional reduction of serious vascular events.[9] The Study of Cardiovascular Events in Diabetes (ASCEND) [10] confirmed the prevention of serious cardiovascular effect from aspirin significantly. However, the benefits accompanied with increase of major bleeding.

A hypercoagulable state with endothelial damage renders patients prone to vascular occlusion, [11] which might relate to microvascular complications of diabetic retinopathy (DR) [12, 13]. Schachat suggested that if a patient had either nonproliferative or mildly proliferative DR, aspirin use appeared safe [14]. Bergerhoff et al. examined randomized controlled clinical trials from the Cochrane Library and Medline in patients with DR comparing aspirin treatment, alone or in combination with dipyridamole, versus placebo. Neither alone nor in combination with dipyridamole did aspirin change the risk of DR [15]. The Early Treatment Diabetic Retinopathy Study (ETDRS) reported that the use of aspirin did not prevent either development of PDR or visual loss [16]. On the other aspect, it did not increase the occurrence of vitreous/preretinal hemorrhages in enrolled patients. They also demonstrated that the severity and duration of these hemorrhages were not significantly affected by the use of aspirin. There were no ocular contraindications to its use (650 mg/d) in patients with diabetes who require aspirin for treatment of cardiovascular disease or other conditions [17]. Recently, The Singapore Epidemiology of Eye Disease (SEED) study revealed that aspirin use is not significantly associated with DR. Rather, aspirin usage may be an indicator of diabetic complications (cardiovascular disease, chronic kidney disease), which often comorbid with severe DR [18]. Conversely, the MADIABETES study demonstrated an association between aspirin use and DR risk in a well-defined cohort of patients with T2DM at a low risk of cardiovascular events [19]. The DAMAD Study, conducted in two French and two UK centers, concluded that antiplatelet agents, aspirin alone (330 mg, three times daily) or in combination with dipyridamole (75 mg, three times daily), significantly slowed the progression of microaneurysm evolution in early DR [20].

Whether using APAC for systemic factors affect DR development or progression is still under debate (supplementary Table 1). In the current study, we revisited this issue and investigated whether the use of APAC therapy affects the development or progression of DR in a Taiwanese longitudinal cohort.

## Methods

### Data source

The Longitudinal Health Insurance Database (LHID) was used for this retrospective cohort study. The LHID is a subset of the National Health Insurance Research Database (NHIRD) covering 23.74 million (99%) Taiwan residents [21]. The details of the LHID have been described in previous studies [22, 23]. Diagnoses in the NHIRD are based on *International Classification of Diseases, Ninth Revision, Clinical Modification* (*ICD-9-CM*) codes. The Ethics Review Board of China Medical University and Hospital in Taiwan approved this study (CMUH-104-REC2-115-R3).

### Study population

We identified patients aged ≥ 20 years with newly diagnosed T2DM (*ICD-9-CM* codes 250.x0 and 250.x2) from January 1, 2000, to December 31, 2010. The index date was defined as the date of the first T2DM diagnosis. We excluded patients with a history of non-proliferative DR (NPDR, *ICD-9-CM* 250.5, 362.01, 362.03–362.06 362.1, 362.81, and 362.82), proliferative DR (PDR, *ICD-9-CM* 362.02, and 379.23), pan-retinal photocoagulation (PRP) treatment, diabetic macular edema (DME, *ICD-9-CM* 362.53, 362.83, and 362.07), or intra-vitreous injection (IVI) treatment before T2DM diagnosis. Two groups were created: one comprised APAC users for T2DM patients on APAC therapy (clopidogrel, aspirin, warfarin, dipyridamole, or ticlopidine) for ≥ 28 days, and APAC nonusers for T2DM patients not on APAC therapy.

### Outcomes, comorbidities, and medications

All patients were followed from the index date until diagnosis of NPDR, PDR, or DME; withdrawal from the insurance program; or December 31, 2011. Baseline comorbidities including hypertension, dyslipidemia, diabetic nephropathy, diabetic neuropathy, heart disease, cardiovascular disease, and peripheral arteriolar disease were identified. Use of medications such as statins, fibrates, and angiotensin-converting-enzyme inhibitors (ACEIs) was also recorded.

### Statistical analysis

Differences in the continuous data of the APAC users and nonusers were tested using Student’s *t* test. Differences in categorical data were analyzed using the chi-square test. The cumulative incidence of NPDR was estimated and plotted for both groups using the Kaplan–Meier method, and the difference was assessed using the log-rank test. The incidence densities of NPDR, PDR, and DME for each group were calculated as the number of NPDR, PDR, and DME events divided by the total person-years. Because some patients may not have taken APAC regularly during the study period, the effect of the drug might be overestimated. Therefore, we considered APAC use as a time-dependent covariate in a Cox proportional hazards model to estimate the effect as HRs and corresponding 95% CIs. Factors significant in the univariate model were adjusted in a multivariate model, namely, age; sex; comorbidities of hypertension, dyslipidemia, diabetic nephropathy, diabetic neuropathy, heart disease, cardiovascular disease, and peripheral arteriolar disease; and medications such as statins and fibrates. We also examined the 5-year risks of PDR and DME after diagnosis of NPDR. All data management and analyses were performed using SAS 9.4 software (SAS Institute, Cary, NC, USA). The significance level was set at *P* < 0.05.

## Results

Table 1 shows a comparison of the baseline characteristics of APAC users and nonusers. APAC users were older on average than nonusers. The proportions of men among the APAC users and nonusers were approximately 53.2% and 51%, respectively. Higher proportions of APAC users were patients with hypertension, dyslipidemia, diabetic nephropathy, diabetic neuropathy, heart disease, cardiovascular disease, and peripheral arteriolar disease and those on statins, fibrates, or ACEIs.

**Table 1:** Distributions of demographic and clinical comorbid variables in study cohorts

	Type 2 DM						
	Anti-platelet/anti-coagulants						
	All (*N* = 73,964)		No (*N *= 64,422)		Yes (*N* = 9542)		
	N	%	n	%	N	%	*P*-value
Age, years							< 0.001
>50	22,758	30.8	18,149	32.6	4609	25.3	
50–64	28,749	38.9	20,875	37.5	7874	43.2	
65+	22,457	30.4	16,722	30.0	5735	31.5	
Mean ± SD ^a^	57.2	13.8	56.8	14.3	58.6	12.1	< 0.001
Sex							< 0.001
Female	35,841	48.5	27,310	49.0	8531	46.8	
Male	38,123	51.5	28,436	51.0	9687	53.2	
Comorbidity							
Hypertension	50,266	68.0	35,537	63.8	14,729	80.9	< 0.001
Dyslipidemia	49,730	67.2	36,311	65.1	13,419	73.7	< 0.001
Diabetic nephropathy	7642	10.3	4776	8.57	2866	15.7	< 0.001
Diabetic neuropathy	1235	1.67	717	1.29	518	2.84	< 0.001
Heart disease	31,067	42.0	20,884	37.5	10,183	55.9	< 0.001
Cardiovascular disease	9649	13.1	6105	11.0	3544	19.5	< 0.001
Peripheral arteriolar disease	6120	8.27	4258	7.64	1862	10.2	< 0.001
Medication							
Statin	35,075	47.4	24,464	43.9	10,611	58.2	< 0.001
Fibrate	22,313	30.2	15,800	28.3	6513	35.8	< 0.001
ACEI	38,899	52.6	27,032	48.5	11,867	65.1	< 0.001

Chi-square test, ^a^ *t* test

**Fig. 1:**
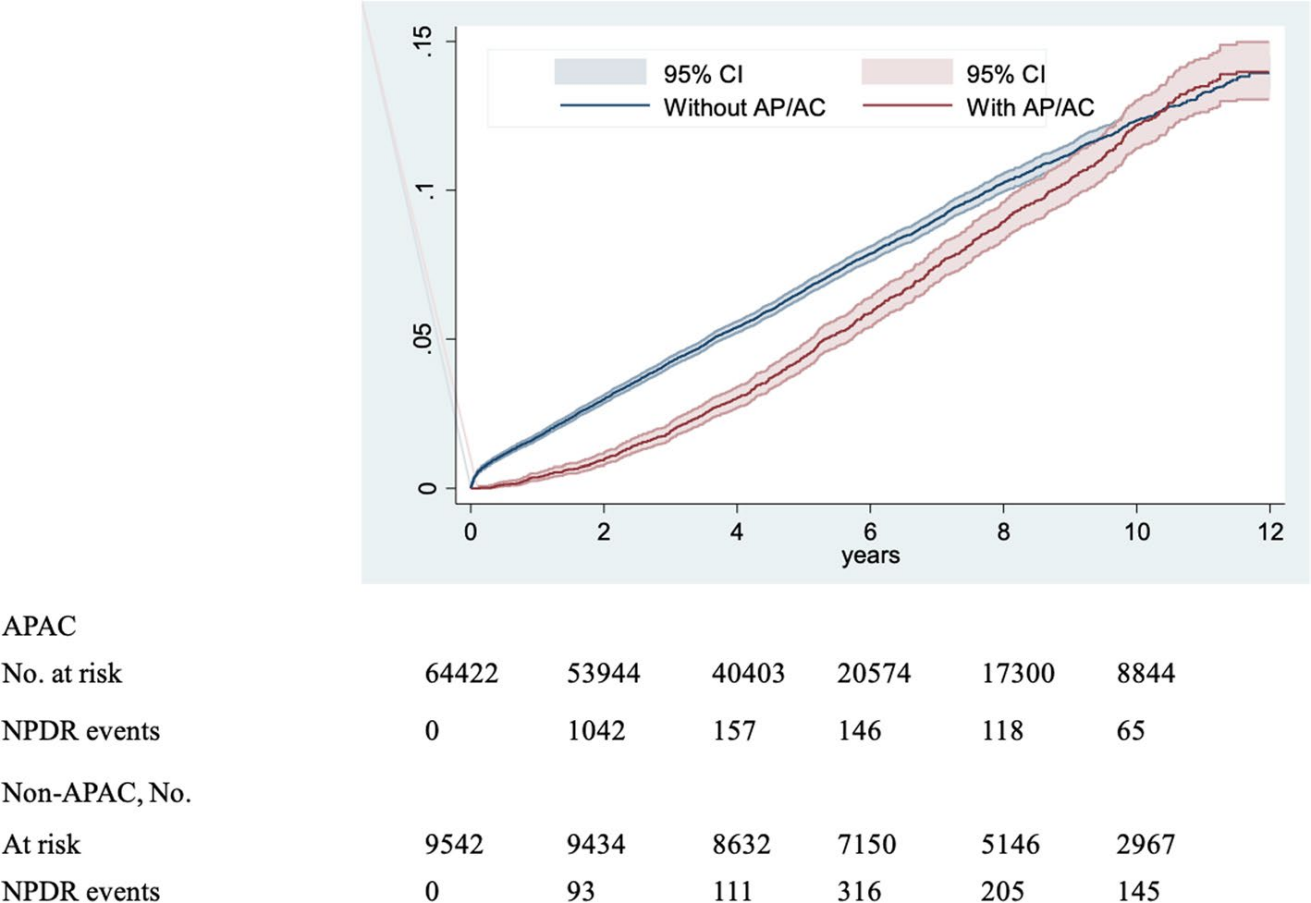
Cumulative incidence of NPDR among APAC users and nonusers with T2DM

The cumulative incidence of NPDR was lower in APAC users than in nonusers (log-rank test *P* < 0.001; Fig. 1). The mean follow-up periods were 7.33 and 5.51 years for APAC users and nonusers, respectively (Table 2). The overall incidence of NPDR was lower in APAC users than in nonusers (10.7 vs. 14.4 per 1000 person-years). APAC users also exhibited a significantly lower risk of NPDR than did nonusers (adjusted HR [aHR] = 0.78, 95% CI = 0.73–0.83).

**Table 2:** Time-dependent regression model results for overall incidence of NPDR, PDR, and DME (per 1000 person-years) and estimated HRs in T2DM patients using and not using APACs

	Anti-platelet/anti-coagulants	
Variables	No (*N* = 55,746)	Yes (*N* = 18,218)
NPDR		
Person-years	307,299	133,609
Follow-up time (y), Mean ± SD	5.51 ± 3.27	7.33 ± 3.16
Event, n	4413	1432
Rate	14.4	10.7
cHR (95% CI)	1 (Reference)	0.75 (0.71, 0.79)***
aHR (95% CI)^a^	1 (Reference)	0.78 (0.73, 0.83)***
PDR		
Event, n	32	13
Rate	0.10	0.09
cHR (95% CI)	1 (Reference)	0.93 (0.49, 1.78)
aHR (95% CI)^a^	1 (Reference)	0.81 (0.42, 1.58)
DME		
Event, n	48	14
Rate	0.15	0.10
cHR (95% CI)	1 (Reference)	0.61 (0.33, 1.10)
aHR (95% CI)^a^	1 (Reference)	0.59 (0.32, 1.08)

Only confounding variables significant in the multivariate model were further analyzed

*Abbreviations*: *aHR* adjusted hazard ratio, *cHR* crude hazard ratio, *CI* Confidence interval, *DME* Diabetic macular edema, *NPDR* Nonproliferative diabetic retinopathy, *PDR* Proliferative diabetic retinopathy, *SD* Standard deviation

**P < 0.05, ***P < 0.001

Table 3 reveals the contributions of various factors to the risk of NPDR as determined by univariate and multivariable Cox proportional hazards models using a time-dependent covariate. The aHR of NPDR increased 1.01-fold (95% CI = 1.01–1.02) for each 1-year age increase. The risk for developing NPDR was higher in patients comorbid with hypertension (aHR = 1.17, 95% CI = 1.09–1.24), diabetic nephropathy (aHR = 1.26, 95% CI = 1.17–1.36), or diabetic neuropathy (adjusted HR = 1.56, 95% CI = 1.34–1.81). The NPDR risk was lower in statin users than in nonusers, with an adjusted HR of 0.74 (95% CI = 0.70–0.78). We also followed up patients for 5 years after they diagnosed with NPDR to observe PDR or DME event occurrences (Table 4). However, PDR and DME risks were not significantly different between APAC users and nonusers. We performed sensitivity analysis to evaluate contributions of each APACs. In monotherapy, only aspirin and Dipyridamole showed significant protection from NDPR development (under Dipyridamole, aHR = 0.85, 95% CI 0.74–0.97, *p* < 0.05). Clopidogrel, Ticlopidine, Warfarin, and Dipyridamole prevented NDPR development in combination with aspirin (aspirin with Clopidogrel, aHR = 0.57, 95% CI 0.44–0.74, *p* < 0.001; aspirin with Ticlopidine, aHR = 0.59, 95% CI 0.39–0.90, *p* < 0.05; aspirin with Warfarin, aHR = 0.59, 95% CI 0.35–0.98, *p* < 0.05; aspirin with Dipyridamole, aHR = 0.61, 95%CI 0.51–0.73, *p* < 0.001) (Table 5).

**Table 3:** HRs and 95% CIs for NPDR and potential risk factors in time-dependent model

Variable	Crude HR (95% CI)		Adjusted HR ^a^ (95% CI)	
APAC	0.75	(0.71, 0.79)***	0.78	(0.73, 0.83)***
Age, years	1.01	(1.01, 1.01)***	1.01	(1.01,1 0.02)***
Sex (vs. Male )	1.05	(1.00, 1.11)*	0.95	(0.90, 1.00)
Comorbidity (vs. no)				
Hypertension	1.07	(1.01, 1.13)*	1.17	(1.09, 1.24)***
Dyslipidemia	0.91	(0.86, 0.96)***	1.06	(0.99, 1.13)
Diabetic nephropathy	1.19	(1.10, 1.28)***	1.26	(1.17, 1.36)***
Diabetic neuropathy	1.47	(1.27, 1.71)***	1.56	(1.34, 1.81)***
Heart disease	0.81	(0.77, 0.86)***	0.77	(0.73, 0.82)***
Cardiovascular disease	0.65	(0.60, 0.72)***	0.61	(0.56, 0.67)***
Peripheral arteriolar disease	0.81	(0.74, 0.89)***	0.81	(0.74, 0.90)***
Medication				
Statin	0.75	(0.71, 0.79)***	0.74	(0.70, 0.78)***
Fibrate	0.91	(0.86, 0.96)***	1.01	(0.95, 1.07)
ACEI	0.99	(0.94, 1.04)	-	-

^a^Only confounding variables significant in the multivariate model were further analyzed.*P < 0.05, **P < 0.01, ***P < 0.001. *Abbreviations*: *ACEI* Angiotensin-converting-enzyme inhibitor, *CI* Confidence interval, *HR* hazard ratio

**Table 4:** Overall PDR and DME events and HRs for PDR and DME measured for T2DM patients with diagnoses of NPDR during 5-year follow-up

	Anti-platelet/anti-coagulants							
	No			Yes				
Variables	Events n	PY	Rate^#^	Events n	PY	Rate^#^	Crude HR (95% CI)	Adjusted HR^†^ (95% CI)
PDR	13	16,146	0.81	4	4658	0.86	1.00 (0.32, 3.06)	0.85 (0.26, 2.77)
DME	21	16,132	1.30	4	4656	0.86	0.62 (0.21, 1.79)	0.65 (0.22, 1.94)

^#^Incidence rate per 1000 person-years, *DME* Diabetic macular edema, *HR* Hazard ratio, *PDR* Proliferative diabetic retinopathy, *PY* Person-years.

^†^Only confounding variables that were significant in the multivariable model were further analyzed.

**Table 5:** Cox proportional hazard regression analysis for the risk of NPDR -associated Aspirin with combined effect of APACS

Variables		N	Event n	Adjusted HR^a^ (95% CI)	*p*-value^#^
Aspirin	Clopidogrel				0.36
No	No	59,329	4704	1(Reference)	
No	Yes	1194	81	0.85(0.68, 1.06)	
Yes	No	12,325	1000	0.82(0.76, 0.88)***	
Yes	Yes	1116	60	0.57(0.44, 0.74)***	
Aspirin	Ticlopidine				0.34
No	No	59,984	4739	1(Reference)	
No	Yes	539	46	1.01(0.76, 1.36)	
Yes	No	13,079	1038	0.81(0.76, 0.87)***	
Yes	Yes	362	22	0.59(0.39, 0.90)*	
Aspirin	Warfarin				0.88
No	No	60,084	4758	1(Reference)	
No	Yes	439	27	0.79(0.54, 1.15)	
Yes	No	13,172	1045	0.81(0.75, 0.87)***	
Yes	Yes	269	15	0.59(0.35, 0.98)*	
Aspirin	Dipyridamole				0.12
No	No	58,000	4559	1(Reference)	
No	Yes	2523	226	0.85(0.74, 0.97)*	
Yes	No	11,534	932	0.83(0.77, 0.89)***	
Yes	Yes	1907	128	0.61(0.51, 0.73)***	

^a^ Model was adjusted for age, sex, comorbidities, and medications

^#^*p*-value for drug interaction

*p < 0.05

***p < 0.001

## Discussion

In our study, APAC users were older, had more comorbidities, experienced more additional diabetic vascular complications, and were more likely to be statin, fibrate, or ACEI users than were APAC nonusers. This result is in accordance with the ATT and ASCEND studies, which have demonstrated that APACs are prescribed to prevent stroke, acute myocardial infarction, and occlusive vascular diseases [9, 10, 24].

Our APAC users had a lower risk of developing NPDR (aHR = 0.78, 0.73–0.83, *p* < 0.001). Use of APACs prevented development of NPDR; however, the protective effects of APACs against PDR and DME were insignificant. Aspirin itself showed significant protective effect against NPDR. Protective effects were even more prominent when aspirin was in combination with other anticoagulants. Dipyridamole monotherapy also showed significant protection. Our results are similar to those of the DAMAD study, which prospectively recruited patients with earlier stages of DR. In the DAMAD study, aspirin alone and aspirin plus dipyridamole reduced microaneurysm formation yearly [20]. However, the dosage of aspirin and dipyridamole in the DAMAD study was two to three times higher than regular clinical prescriptions. In a 5-year animal study, aspirin significantly inhibited early stages of DR, including the development of retinal hemorrhages and acellular capillaries, but had limited effects on other pathological changes [25]. Conversely, the prospective MADIABETES study from Spain observed that the risk of DR increased 1.65 times with aspirin use, after adjustments for confounders [19]. The SEED study, after adjusting for covariates, demonstrated that aspirin use was significantly associated with vision-threatening DR; however, the association decreased after adjustments for other diabetic complications, including cardiovascular disease and chronic kidney disease [18]. The ETDRS, which included patients with mild to severe NPDR and PDR, found no significant effects of aspirin on DR development or progression [16]. Moreover, aspirin neither prevented visual loss nor increased hemorrhage rates in DR patients [26]. The dosage of aspirin in the ETDRS was higher than the typically prescribed dose. In study from Italy, aspirin was associated with higher hazard ratio of DR and PDR, but significance disappeared after adjustment with non-fatal major adverse cardiovascular events and diabetic kidney disease [27]. Ticlopidine slowed down microaneurysm progression in insulin-treated diabetic patients in a 3-year follow-up in TIMAD study [28]. In another Belgian study, there was a more favorable trend toward Ticlopidine group however insignificant, which might due to limited study number [29]. In our study, the effects from Clopidogrel, Ticlopidine, and Warfarin were insignificant when prescribed solely. However, the numbers of these APACs were much smaller compared with aspirin and Dipyridamole. Our results also demonstrated that APAC therapy neither increases nor reduces the risk of PDR or DME. However, the event number in our study of PDR or DME were limited and further investigations are needed to explore effects from APAC.

In our cohort, older age, hypertension, and diabetic microvascular angiopathies of nephropathy and neuropathy were associated with higher risks of NPDR; however, patients with cardiovascular disease or peripheral occlusive arteriolar disease had lower risks. The effect of age is inconsistent. Older age was a risk factor for DR in studies in South Korea and Saudi Arabia [30, 31]. In the United Kingdom Prospective Diabetic Study (UKPDS), older age was a risk factor for the progression but not for the incidence of DR [32]. The WESDR, however, reported younger age as a risk factor, [33] and the 10-year incidence of DR, progression of DR, and progression to PDR were the highest in the under-30 age group with diabetes [34]. The ETDRS also reported younger age as a risk factor for PDR and severe visual loss [35].

Associations between DR and diabetic neuropathy and diabetic nephropathy have been identified [35, 36]. Kotlarsky et al. observed that nephropathy preceded retinopathy in their study [37]. Another study indicated that hypertriglyceridemia may be a surrogate marker of DME [38]. The effects of fibrates and statins vary; however, the certainty of relevant evidence appeared low in a systemic review [39]. In the Action in Diabetes and Vascular Disease: Preterax and Diamicron Modified-Release Controlled Evaluation (ADVANCE) and its subsequent studies (ADVANCE-on), lower-extremity ulceration or amputation increased the risk of vision-threatening DR [40]. Klein et al. found significant associations between DR and coronary heart disease, stroke, and gross proteinuria [41]. Considerable coronary artery calcification in adults with chronic type 1 DM was associated with retinopathy [42]. Our study, however, showed that these macrovascular diseases were inversely related to the risk of DR. Several explanations are possible. First, positive correlations are more frequently reported than negative ones. Second, blood pressure and blood sugar are important controllable factors affecting DR development. Systemic factors may be strictly controlled after macrovascular complications. In the Wisconsin Epidemiologic Study of Diabetic Retinopathy (WESDR), which focused on type 1 diabetes, the progression rate of DR in 25 years was 83%, in which higher glycosylated hemoglobin [HbA(1 C)] level, and increase diastolic blood pressure were aggravating factors [43]. From UK database, which focused on type 2 DM, increase of HbA(1 C) and systolic blood pressure increased the odds [44]. In UKPDS, incidence and progression were strongly associated with hyperglycemia [32].

Duration is another nonmodifiable but influential factor. In type 2 diabetes, retinopathy was concomitantly diagnosed with diabetes in about one-fifth of patients [32, 44]. The rate of development of DR increases when duration increases, and prevalence increases amongst patients with diabetes exceeding 10 years [45]. Our cohort comprised newly diagnosed type 2 diabetes from January 1st, 2000, to December 31st, 2010, and the follow-up duration was 12 years. The strength of our cohort in Taiwan has up to 99% of national health insurance coverage, which reflected the panorama. There are several limitations in our study. First, this is a retrospective study, and that data on clinical measurements, such as blood pressure and blood sugar control, could not be retrieved from the database. Second, patients of APAC use with more than 28 days were included. Compared with the long duration of follow-up to 12 years, it was a short period of treatment. Third, the dosage effect from different APACs was not analyzed in this study. Fourth, precise duration of hyperglycemia is hard to track in type 2 diabetes. Hyperglycemia is asymptomatic and lurks before complications develop. In our cohort, the patients were included at diagnosis of type 2 diabetes and tracked until DR development. This is compatible with the real-world clinical situations, and DR development increased when the duration increased as reflected in Fig. 1.

APACs show beneficial effects on NPDR development in diabetics. The effect of APAC on progression from NPDR to DME or PDR was not significant from our study. However, it is prudent to draw conclusion from our study since the number was too small. Further investigation is needed to explore the effect of APAC on development and progression to PDR and DME.

## Conclusions

APAC medications have a protective effect against NPDR development. Diabetic patients benefit from single use of aspirin or dipyridamole on prevention of NPDR.

## Supplementary Information

**Additional file 1: Table S1**. Studies of APAC use.

## Data Availability

All data and materials analyzed in this study are available at corresponding author.

## Abbreviations

APAC: Antiplatelet/anticoagulant
CI: Confidence interval
DM: Diabetes mellitus
DME: Diabetic macular edema
DR: Diabetic retinopathy
HR: Hazard ratio
LHID: Longitudinal Health Insurance Database
NHIRD: National Health Insurance Research Database
NPDR: Non-proliferative diabetic retinopathy
PDR: Proliferative diabetic retinopathy
T2DM: Type 2 diabetes mellitus
