# Supplementary material for: Effect of anticoagulant/antiplatelet therapy on the development and progression of diabetic retinopathy
Source: BMC Ophthalmol. 2022 Mar 17;22:127. doi: 10.1186/s12886-022-02323-z (PMC8932222; doi:10.1186/s12886-022-02323-z)
Supplement: Supplementary file 1 — Additional file 1: Table S1. Studies of APAC use. [file 12886_2022_2323_MOESM1_ESM.docx]

| Supplementary Table 1. Studies of APAC use | | | | | |
| --- | --- | --- | --- | --- | --- |
| Study name  Authors, country (year) | Study design | Patients number | APAC dosage | Results | Conclusion |
| SEED study  Yaun Shi et al., Singapore [19] | Population-based, cross sectional | 2061 |  | Significance between Aspirin and either DR or VTDR use decreases after adjustment of CVD and CKD. | Aspirin use was not significantly associated with DR but might be an indicator of diabetic complications (CVD, CKD) that were co-present with more severe DR type. |
| MADIABETES  Miguel Á. Salinero-Fort, et al.  Madrid, Spain [20] | Prospective, 4-year | 3,443 type 2 DM patients |  | Aspirin use is a significant risk factor for the incidence of diabetic retinopathy (aHR=1.65, HR 95% CI 1.22-2.24, P<0.001). | The study demonstrates an association between aspirin use and diabetic retinopathy risk in a well-defined cohort of patients with Type 2 Diabetes Mellitus at low risk of cardiovascular events. |
| The DAMAD Study Group, French-UK [21] | double-blind randomized controlled clinical trial  follow-up three years | 420 patients with early DR | Aspirin alone (330 mg 3 times daily), or aspirin alone (330 mg 3 times daily) in combination with dipyridamole (75 mg 3 times daily), versus placebo | There was no significant difference between the aspirin-alone group (*n* = 145) and the aspirin-plus-dipyridamole group (*n* = 142). In the placebo group (*n =* 133) the mean yearly increase of MAs was significantly higher than in the treated group (P = .02,1-tailed *t* test). | Aspirin alone and in conjunction with dipyridamole significantly slows the progression of MA evolution in early diabetic retinopathy. |
| Early Treatment Diabetic Retinopathy Study Research Group [27] | ETDRS study | 3711 | Aspirin (650mg daily) or placebo | with mild-to-severe non-proliferative or early proliferative diabetic retinopathy | Aspirin has no clinically important beneficial effects on the progression of retinopathy |
| Pia Clara Pafundi, et al. Italy  NO BLIND study [28] | Cross-sectional, multicenter, observational study | 2068 | 100 mg/day orally or dosage usually taken in Italy | Significance between DR and aspirin disappeared after adjusting with major adverse cardiovascular effects and diabetic kidney disease. | There is lack of any independent association between aspirin use and DR, particularly with PDR. |
| TIMAD  France [29] | A randomized, double-masked, placebo-controlled trial,  3 years | 435 type 2 DM patients, with insulin treated and non-insulin treated | Ticlopidine 500 mg daily | Ticlopidine was significantly beneficial to insulin-treated diabetic patients, inducing a sevenfold reduction of the yearly microaneurysm progression (0.23$\pm$6.66) compared with the placebo (1.57 $\pm$5.29) (p$=$.03). | Ticlopidine slows down the progression of non-proliferative diabetic retinopathy in insulin treated patients. |
| BTRS  Belgian [30] | Multi-center, double blind | 100 insulin-treated diabetics with BDR | Ticlopidine 250mg, b.i.d | The proportion of pa­tients with favorable results was higher in the ticlopidine group (55.2%) than in the placebo group (36.6%). However, this difference did not reach statistical significance (p = 0.123). | Ticlopidine only gave a promise towards the slowing down of back­ground diabetic retinopathy. |
| DR: diabetic retinopathy; VTDR: vision threatening diabetic retinopathy; BDR: background diabetic retinopathy; CVD : cardiovascular disease; CKD: chronic kidney disease; DM: diabetic mellitus; MA: microaneurysm; aHR: adjusted hazard ratio; HR: hazard ratio; CI: confidence interval; FAG: fluorescein angiograms | | | | | |
